# The intersection of sexually transmitted infections and antimicrobial stewardship: opportunities for synergy

**DOI:** 10.1017/ash.2026.10754

**Published:** 2026-07-22

**Authors:** Aditi Ramakrishnan, Sheetal Kandiah, Shawnalyn Sunagawa, Jonathan Ryder

**Affiliations:** 1 Division of Infectious Diseases, Department of Medicine, Washington University School of Medicine in Saint Louis, St. Louis, MO, USA; 2 Bursky School of Public Health, Washington University in St. Louis, St. Louis, MO, USA; 3 Division of Infectious Diseases, Department of Medicine, Emory University School of Medicine, Atlanta, GA, USA; 4 Division of Infectious Diseases, Department of Internal Medicine, University of Nebraska Medical Center, Omaha, NE, USA

## Abstract

Following the introduction of doxycycline post-exposure prophylaxis (doxy PEP), concerns regarding associated antimicrobial resistance have emerged. In this piece, we discuss a range of collaborative strategies between the historically siloed fields of antimicrobial stewardship and sexually transmitted infections to guide the stewardship of doxy PEP and diagnostics for STI care.

Doxycycline post-exposure prophylaxis (doxy PEP) has been shown to significantly decrease the incidence of chlamydia, syphilis, and gonorrhea among men who have sex with men and transgender women when taken within 72 hours after condomless sex.^1–3^ In 2024, the Centers for Disease Control recommended doxy PEP as a patient-managed biomedical sexually transmitted infection (STI) prevention strategy for these populations.^4^ However, key concerns regarding antimicrobial resistance (AMR) have emerged.^5^ Additionally, multiple provider specialties are now prescribing doxy PEP, with unclear levels of monitoring patient utilization, AMR, and STI screening. In this piece, we discuss antimicrobial and diagnostic stewardship strategies with a focus on doxy PEP use and STI diagnostic utilization to improve overall STI care.

AMR associated with doxy PEP use has been observed in recent clinical trials (Table 1). The Doxy PEP study demonstrated a higher frequency of tetracycline-resistant gonorrhea among doxy PEP recipients who had a *Neisseria gonorrhea* culture available.^2^ Additionally, while the doxy PEP arm had lower overall *Staphylococcus aureus* carriage, doxycycline-resistant *Staphylococcus aureus* colonization increased. A surveillance cohort demonstrated an associated increase in tetracycline-resistant gonorrhea after the introduction of doxy PEP and increased colonization with tetracycline-resistant *Staphylococcus aureus*.^6^ While the clinical significance of these observations is still unclear and more robust real-world data is needed, as doxy PEP use expands, associated AMR is expected. These findings suggest the need to carefully integrate traditional antimicrobial and diagnostic stewardship approaches with STI care (Figure 1).

**Figure 1.**
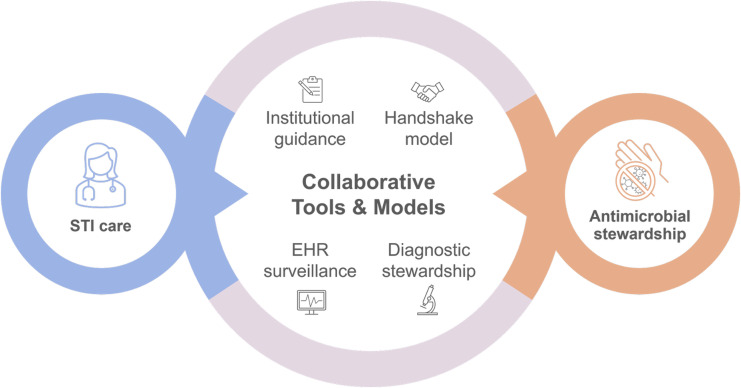
Collaborative tools and models integrating the fields of sexually transmitted infections/sexual health and antimicrobial and diagnostic stewardship. Such tools and models can include. 1) Institutional antibiotic prescribing guidance, 2) Handshake models to promote consistent prescribing and STI testing across provider specialties in the outpatient and inpatient environments, 3) EHR surveillance of doxy PEP use, antimicrobial resistance, and STI screening, and 4) Diagnostic stewardship elements including specific order sets, educational initiatives, and clinical decision-support systems.

**Table 1.** Summary of clinical studies examining changes in antimicrobial resistance in non-sexually transmitted infections associated with doxy PEP use Table 1 long description.

Study name (location; year)	Study design (N)	Bacteria and culture type	Effect of doxy PEP on antimicrobial resistance
ANRS 174 DOXYVAC^1^ (Paris, France; 2024)	RCT (n = 556)	MRSA from pharyngeal colonization swabs ESBL *Escherichia coli* from rectal colonization swabs	No significant difference in percentage of participants in doxy PEP and standard of care arms with MRSA (*P* = .83) or ESBL *E. coli* colonization (*P* = .5) No measurements of tetracycline resistance
Doxy PEP ^2,12,13^ (Seattle and San Francisco, USA; 2025)	RCT (n = 637) Sub-Group of RCT (n = 150)	*Staphylococcus aureus* from oro-nasopharyngeal colonization swabs DNA-seq and RNA-seq of rectal swabs to evaluate gut microbiome and antimicrobial restistome	Doxycycline-resistant *S. aureus* increased from 5% at baseline to 15% at 12 months (*P* < .001) in doxy PEP group, whereas in standard of care there was no significant change (11% to 5%, *P* = .179) Doxy PEP associated with increase in doxycycline-resistant *S. aureus* (HR 3.89, 95% CI 1.42–10.68, *P* = .0044) among those without baseline doxycycline resistance Doxy PEP associated with expansion of tetracycline antimicrobial resistance genes in the resistome. Doxy PEP did not meaningful change the taxonomy of the microbiome, alpha diversity, beta diversity or microbiome mass
King County Sexual Health Clinic^6^ (Seattle, USA; 2025)	Surveillance Cohort (n = 838 for *S. aureus*, n = 512 for GAS)	*S. aureus* and GAS nares and pharyngeal colonization swabs	Tetracycline-resistant *S. aureus* more common in doxy PEP users than non-users (18% vs 8%, *P* < .0001) GAS colonization more common in doxy PEP users than non-users (9% vs 4%, *P* = .008) but tetracycline resistance was similar (80% vs 85%, *P* = 1.0)

DNA-seq, metagenomic DNA sequencing; doxy PEP, doxycycline pre-exposure prophylaxis; ESBL, extended-spectrum beta-lactamase; GAS, Group A *Streptococcus*; MRSA, methicillin-resistant *Staphylococcus aureus*; RCT, randomized controlled trial; RNA-seq (metatranscriptomic RNA sequencing); SOC, standard of care.

One clear opportunity for collaboration between STI and stewardship teams is through the development of institutional antibiotic prescribing guidance and associated models of care to ensure consistent prescribing behavior across diverse provider specialties. For example, based on the above observations of AMR, teams can collaborate to integrate reviews of appropriateness, local antibiograms, and antibiotic duration to develop thoughtful clinical guidance regarding treatment for CAP and SSTI among individuals taking doxy PEP.^6^ Such guidance can integrate stewardship and STI care viewpoints through providing step-by-step frameworks for clinicians to engage in conversations grounded in shared decision-making with patients regarding doxy PEP and associated antibiotic treatment decisions. As providers of varying specialties are prescribing doxy PEP, STI services can employ the “handshake model” from antimicrobial stewardship best practices to improve consistent provider prescribing behavior across diverse inpatient and outpatient specialties.^7^ For example, pharmacists trained in stewardship could provide audit and feedback to providers regarding doxy PEP practices in ambulatory care or emergency department settings. While the handshake model has been traditionally applied in the inpatient setting, extending the model to supporting doxy PEP prescribing practices in this manner could catalyze implementation of this key stewardship element in the outpatient context.

Antimicrobial stewardship programs can also provide frameworks and tools to shape doxy PEP prescribing and associated STI testing. These can include key elements of antimicrobial stewardship programs such as standardized prescribing pathways, electronic health record (EHR) order sets, and streamlined antibiotic monitoring protocols.^8^ Since many institutional antimicrobial stewardship programs interface with both inpatient and ambulatory settings, they can leverage the alignment of these EHR systems to implement such tools to further support prescribing consistency. For example, stewardship programs can leverage the EHR to monitor long-term doxy PEP use, signal to providers if individuals receiving doxy PEP are not receiving guideline-directed STI screenings, and prompt providers to recommend STI screening at all anatomical sites of exposure, if relevant to individuals, as such “three-site” screening recommendations are not optimized even in STI clinics.^9^ Antimicrobial stewardship programs can also leverage their relationship with clinical microbiology departments to further coordinate surveillance of potential doxy PEP-associated resistance patterns. This can enable systems to better balance individual patient benefits with broader public health considerations. A strength of antimicrobial stewardship programs is to collaborate across specialties and disciplines, such as microbiology and information technology (IT), which in turn can facilitate health systems’ implementation and monitoring of doxy PEP in an appropriate, safe, and effective manner.

Apart from doxy PEP use, another opportunity for STI care teams and stewardship programs to interface is in the realm of diagnostic stewardship: ensuring the right test for the right patient at the right time. Diagnostic stewardship programs are equipped with unique tools such as order set creation/implementation, educational initiatives, and clinical decision-support implementation that can be leveraged for STI screening in inpatient and outpatient settings. These interventions can encourage clinicians to choose the best tests (eg, HSV PCR for symptomatic lesions) while discouraging or even restricting low-yield testing (eg, HSV antibodies for screening) to follow best practices. For example, implementation of provider education and an EHR smart phrase was associated with increased STI screening among people who inject drugs with serious injection-related infections.^10^ Such interventions can emerge particularly through interdisciplinary collaboration between STI, stewardship, microbiology, and IT teams.

An emerging area in STI diagnostics is the growing availability of multiplex PCR panels and next-generation sequencing tests. Multiplex PCR panels, which are increasingly ordered to evaluate symptoms of vaginitis and include *Candida*, bacterial vaginosis, and *Trichomonas* testing, provide useful information when testing symptomatic patients. However, efforts to discourage testing and treatment of asymptomatic patients, particularly individuals with positive *Candida* or bacterial vaginosis targets, can be improved. Additionally, next-generation sequencing testing often detect organisms of dubious pathogenicity (eg, *Mycoplasma hominis* or *Ureaplasma urealyticum*) or AMR genes of unclear significance. Another application of molecular testing is PCR detection of fluoroquinolone resistance in *Neisseria gonorrhea*, which has the potential to help identify patients for whom more targeted therapy may be beneficial.^11^ Diagnostic stewardship and STI care teams can coordinate to monitor utilization of these tests, create guidelines for appropriate use, educate clinicians, and implement laboratory restrictions regarding test use. Providing clear guidance on appropriate tests for specific clinical situations and indications for specialist referral can help improve diagnostic yield and reduce unnecessary testing and antibiotic overtreatment of commensal organisms.

The recommendation of doxy PEP as a prevention tool has ushered in a new era for potential synergy between the historically siloed fields of STI care and antimicrobial stewardship. Strategies such as collaboration on institutional guidance regarding antibiotic use for individuals taking doxy PEP, application of the stewardship “handshake model” to improve provider consistency in STI prescribing and testing patterns, and leveraging stewardship EHR programs to facilitate stewardship of doxy PEP and STI diagnostic tests can create a strong interdisciplinary platform to ensure appropriate antibiotic prescribing and diagnostic testing. While recommendations and implementation of doxy PEP has varied globally,^14–16^ such collaborative strategies bridging ASP and sexual healthcare can extend to under-resourced settings in both developed and developing countries.

While AMR may increase due to widespread doxy PEP adoption, clinical trial and real-world utilization data demonstrate a clear benefit to the individual patient and associated sexual networks regarding reduction in STI incidence.^1–3,6^ Various interventions such as penicillin prophylaxis for recurrent cellulitis, suppressive antimicrobials for retained prosthetic joint infections, or utilization of long-term antimicrobials for non-infectious disease conditions, such as doxycycline for acne and rosacea treatment, may have similar downstream consequences. However, since doxy PEP is still a novel intervention, we have a rich opportunity to integrate antimicrobial and diagnostic stewardship with STI management—the time is now.

## Table 1. Long description

The table has four rows and five columns. The columns are labeled Study name (location; year), Study design (N), Bacteria and culture type, and Effect of doxy PEP on antimicrobial resistance. The rows present data for three studies: ANRS 174 DOXYVAC, Doxy PEP, and King County Sexual Health Clinic. Each row includes details on the study location and year, study design and sample size, types of bacteria and cultures used, and the effects of doxy PEP on antimicrobial resistance. Notable findings include no significant difference in MRSA and ESBL E. coli colonization in the ANRS study, increased doxycycline-resistant S. aureus in the Doxy PEP study, and higher tetracycline-resistant S. aureus in doxy PEP users in the King County study.

Navigate back to Table 1..
